# The odd log-logistic generalized exponential distribution: Application on survival times of chemotherapy patients data

**DOI:** 10.12688/f1000research.127363.2

**Published:** 2023-08-10

**Authors:** Arnold K. Fulment, Srinivasa Rao Gadde, Josephat K. Peter

**Affiliations:** 1 Mathematics and Statistics, The University of Dodoma, Dodoma, 338, Tanzania

**Keywords:** Odd log-logistic generalized exponential distribution, maximum-likelihood estimation, generating functions, moments, simulation and order statistics.

## Abstract

**Background**: The creation of developing new generalized classes of distributions has attracted applied and theoretical statisticians owing to their properties of flexibility. The development of generalized distribution aims to find distribution flexibility and suitability for available data. In this decade, most authors have developed classes of distributions that are new, to become valuable for applied researchers.

**Methods**: This study aims to develop the odd log-logistic generalized exponential distribution (OLLGED), one of the lifetime newly generated distributions in the field of statistics. The advantage of the newly generated distribution is the heavily tailed distributed lifetime data set. Most of the probabilistic properties are derived including generating functions, moments, and quantile and order statistics.

**Results:** Estimation of the model parameter is done by the maximum likelihood method. The performance of parametric estimation is studied through simulation. Application of OLLGED and its flexibilities is done using two data sets and while its performance is done on the randomly simulated data set.

**Conclusions**: The application and flexibility of the OLLGED are ensured through empirical observation using two sets of lifetime data, establishing that the proposed OLLGED can provide a better fit in comparison to existing rival models, such as odd generalized log-logistic, type-II generalized log-logistic, exponential distributions, odd exponential log-logistic, generalized exponential, and log-logistic.

## 1. Introduction

To cover the need for applied statistics in a field like economics, education, engineering, geology, health, and many others to mention, as well as in the area of development of models and analysis for lifetime data, some statistical probability distributions have been developed. However, these developed distributions have not been able to suffice the whole vacuum of data fit. As a result, room for the development of new distributions by researchers to model day-to-day lifetime data has always been there. The creation of developing new generalized classes of distributions has attracted applied and theoretical statisticians owing to their properties of flexibility. The development of generalized distribution aims to find distribution flexibility and suitability for available data. In this decade, most authors have developed classes of distributions that are new, to become valuable for applied researchers. Development methods for the new distribution are numerous in the literature. Generalization of probability distributions was initially introduced ^1^ where the authors generalized Weibull probability distribution, and the result was named exponential Weibull distribution which is common in modeling lifetime data. ^2^ Later, a modeling failure time data was developed ^3^ by Lehmann-type alternatives named as an exponentiated form to base distribution. Later on, two parameters of generalized exponential distribution (GED) were developed, ^4^ also called exponential distribution (ED). For more details on GED, refer to Refs. 5, 6. Due to its importance in statistical inference and reliability applications, numerous authors studied the various properties of this distribution. ^5^^,^^7^^–^^14^ It is proved that the GED is an excellent substitute for gamma, log-Normal and Weibull distributions.

The motive for extending distributions for modeling lifetime data is the capacity to simulate both monotonically and non-monotonically growing, decreasing, and constant failure rates, or more critically with bathtub shaped failure rates, even if the baseline failure rate is monotonic. The fundamental justifications for implementing a new distribution model in practice are as follows: to create tail weight distributions for modeling various real data sets, to generate distributions with negative, positive, and symmetric skewness, to define special models with all varieties of hazard rate functions, to make the kurtosis more flexible than the baseline distribution, and to consistently produce better fits than other generated distributions with the same underlying model.

A random variable *X* is said to have the GED, hereafter referred to as baseline distribution with shape $\left(\alpha \right)$ and scale $\left(\lambda \right)$ parameters if its probability density function (PDF) and cumulative density function (CDF) are given as respectively:

$$f\left(x\right)=\alpha \lambda e^{-\mathrm{\lambda x}}{\left(1-e^{-\mathrm{\lambda x}}\right)}^{\alpha -1};\lambda >0,x\geq 0,\alpha >0$$ (1)

$$F\left(x\right)={\left(1-e^{-\mathrm{\lambda x}}\right)}^{\alpha }.$$ (2)

On the other hand, generalization was done in beta distribution under the name of generalized beta distribution; for more details refer to Ref. 15. They developed further generalized beta-generated (GBG) distribution, with a total of three parametric values. ^16^ There are other many generalization methods in the literature depending on the nature of the distribution of data in hand. ^17^ The researchers intend to introduce a new family of distribution which is named odd log-logistic generalized exponential distribution (OLLGED) to model heavy-tailed data set in daily-to-daily data set. ^18^

The OLLGED is a generalization of exponential distribution with the addition of two parameters, which makes it have a total of three parametric values. The proposed distribution has a total of three parameters, lambda ( λ ) as the only scale parameter, alpha ( α ) and gamma ( γ ), which are shape parameters introduced by generalization methods procedures, making it more flexible and thus, enabling the OLLGED to have an application to lifetime data and more extended to acceptance sampling plans and quality control charts. ^19^^,^^20^

This paper is aimed at studying and defining a new lifetime paradigm namely OLLGED. Wide-ranging statistical properties and its applications through real data sets are given. More works on OLLGED have been presented. ^21^^,^^22^ The distribution proposed contains several lifetime distributions, such as GED. ^23^^–^^25^ OLLGED was introduced here for the reason:

1. It comprises a number above mentioned of well-known lifetime particular distributions;
2. The OLLGED demonstrates that shapes of hazard rates as monotonically decreasing, increasing, J, reversed-J, bathtub, and upside-down bathtub, which establishes that the recommended model has advanced to other lifetime distributions in hand;
3. To construct distribution to be used in special models that are capable of modeling skewed life time data and can also be used in a various areas of applications;
4. From the studies in section 2, the OLLGED would be considered with GED as baseline distribution ^6^;
5. Asymmetric data that may not be well-fitted to other regular distributions may be fitted properly by the proposed model; and
6. The OLLGED beats numerous competitor distributions based on two real data illustrations.

The class of distributions called the OLL-G family (generalized log-logistic-G family) by adding one more shape parameter was introduced. ^22^ OLL-G family PDF and CDF are as follows:

$$g\left(x\right)=\frac{\mathrm{\gamma f}\left(x\right){\left\{F\left(x\right)\left[1-F\left(x\right)\right]\right\}}^{\gamma -1}}{{\left\{F{\left(x\right)}^{\gamma }+{\left[1-F\left(x\right)\right]}^{\gamma }\right\}}^{2}}\;$$ (3)

$$G\left(x\right)=\frac{F{\left(x\right)}^{\gamma }}{F{\left(x\right)}^{\gamma }+{\left[1-F\left(x\right)\right]}^{\gamma }}\;$$ (4)

We note that

$$\gamma =\frac{\log\left[\frac{G\left(x\right)}{\bar{G}\left(x\right)}\right]}{\log\left[\frac{F\left(x\right)}{\bar{F}\left(x\right)}\right]}.$$

The next sections of this article are organized as follows; in Section 2, special models associated with OLLGED are explained. In Section 3, useful expansions and OLLGED properties are derived. Section 4 discussed the estimations of the parameters. The simulation study is carried out based on various parametric values of the proposed distribution in Section 5. Data analysis is done using two-lifetime data sets in Section 6, and in Section 7 of the article, discussion and conclusion are done.

## 2. The OLLGED and its special models

Using equations (1) and (2) in equations (3) and (4), we can develop the OLL-G family with baseline distribution as GED and it is named OLLGED. The PDF and CDF of OLLGED are given by

$$g\left(x\right)=\frac{\gamma \alpha \lambda e^{-\mathrm{\lambda x}}{\left(1-e^{-\mathrm{\lambda x}}\right)}^{\alpha -1}{\left\{{\left(1-e^{-\mathrm{\lambda x}}\right)}^{\alpha }\left[1-{\left(1-e^{-\mathrm{\lambda x}}\right)}^{\alpha }\right]\right\}}^{\gamma -1}}{{\left\{{{\left(1-e^{-\mathrm{\lambda x}}\right)}^{\alpha }}^{\gamma }+{\left[1-{\left(1-e^{-\mathrm{\lambda x}}\right)}^{\alpha }\right]}^{\gamma }\right\}}^{2}}\;$$ (5)

$$G\left(x\right)=\frac{{{\left(1-e^{-\mathrm{\lambda x}}\right)}^{\alpha }}^{\gamma }}{{{\left(1-e^{-\mathrm{\lambda x}}\right)}^{\alpha }}^{\gamma }+{\left[1-{\left(1-e^{-\mathrm{\lambda x}}\right)}^{\alpha }\right]}^{\gamma }};x>0,\lambda >0,\alpha >0,\gamma >0.$$ (6)

Here γ and α are shape parameters and λ is a scale parameter of the distribution. Henceforth, if a random variable X follows to OLLGED with shape parameters $\left(\gamma ,\alpha \right)$ and scale parameter λ , it is denoted as $X∼\text{OLLGED}\left(\gamma ,\alpha ,\lambda \right)$ .

The OLLGED is a more flexible distribution that provides several distributions by inter-changing parametric values. It contains the following models:

i) When γ=1 , the resulting distribution becomes GED. ^6^
ii) When α=1 , the resulting distribution becomes an OLLGED.
iii) When γ=1 and α=1 , the resulting distribution becomes an ED.

**Figure 1.**
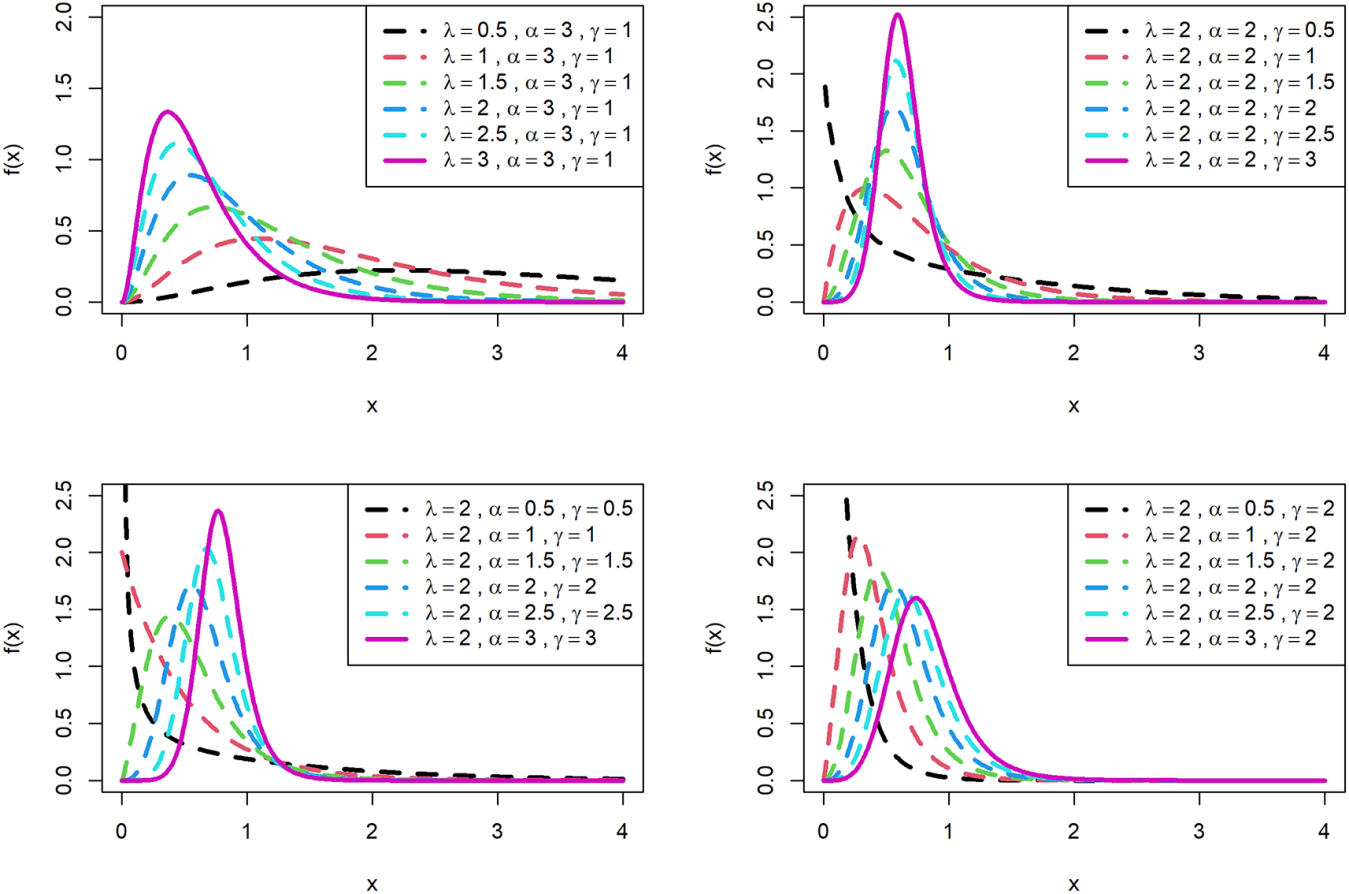
Visual presentation of pdf plots of the OLLGED for various parameters.

**Figure 2.**
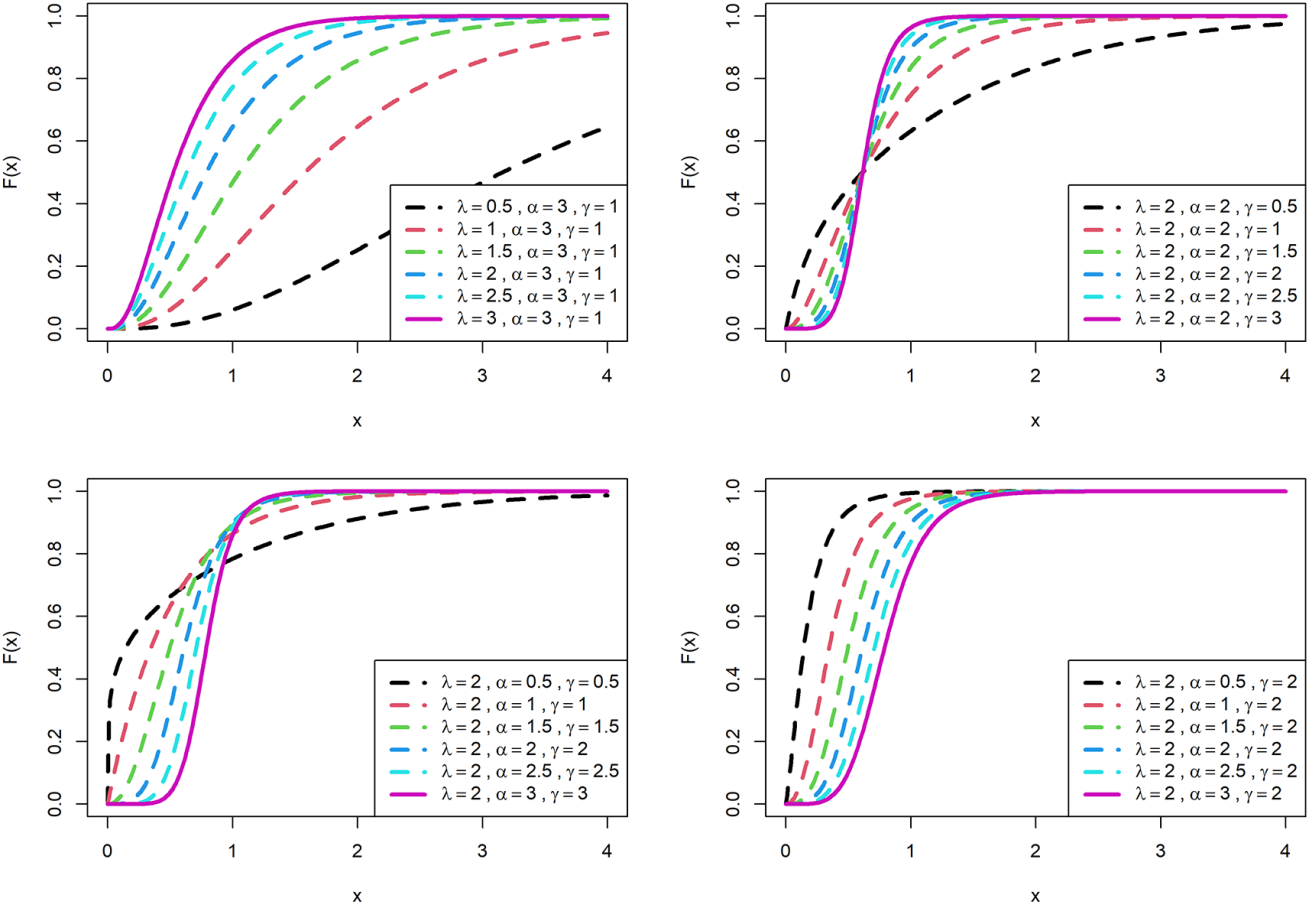
Visual presentation of CDF plots of the OLLGED for various parameters.

Figure 1 is displayed for PDF and Figure 2 is displayed for CDF for various parametric values for OLLGED. Figures 1 and 2 reveal that the OLLGE family produces distributions with different shapes namely symmetrical, reversed-J, and left and right-skewed. Figures 1 and 2 revealed that the OLLGED is more flexible with different shapes namely symmetrical, Reversed-J, and left and right-skewed. Figures 1 and 2 revealed that the OLLGED is more flexible with various parameter values considered which gives the property that it was suitable to use for lifetime data, for whichever data set distribution will fit its characteristics. More specifically, when γ≤1 and α≤1 the shape of the distribution is reversed-J.

The survival function and hazard rate, $s\left(x\right)$ and $h\left(x\right)$ respectively for OLLGED are respectively given below:

$$s\left(x\right)=\frac{{\left[1-{\left(1-e^{-\mathrm{\lambda x}}\right)}^{\alpha }\right]}^{\gamma }}{{{\left(1-e^{-\mathrm{\lambda x}}\right)}^{\alpha }}^{\gamma }+{\left[1-{\left(1-e^{-\mathrm{\lambda x}}\right)}^{\alpha }\right]}^{\gamma }}$$ (7)

$$h\left(x\right)=\frac{\gamma \alpha \lambda e^{-\mathrm{\lambda x}}{\left(1-e^{-\mathrm{\lambda x}}\right)}^{\alpha \gamma -1}{\left\{1-{\left(1-e^{-\mathrm{\lambda x}}\right)}^{\alpha }\right\}}^{-1}}{{{\left(1-e^{-\mathrm{\lambda x}}\right)}^{\alpha }}^{\gamma }+{\left[1-{\left(1-e^{-\mathrm{\lambda x}}\right)}^{\alpha }\right]}^{\gamma }}\;$$ (8)

**Figure 3.**
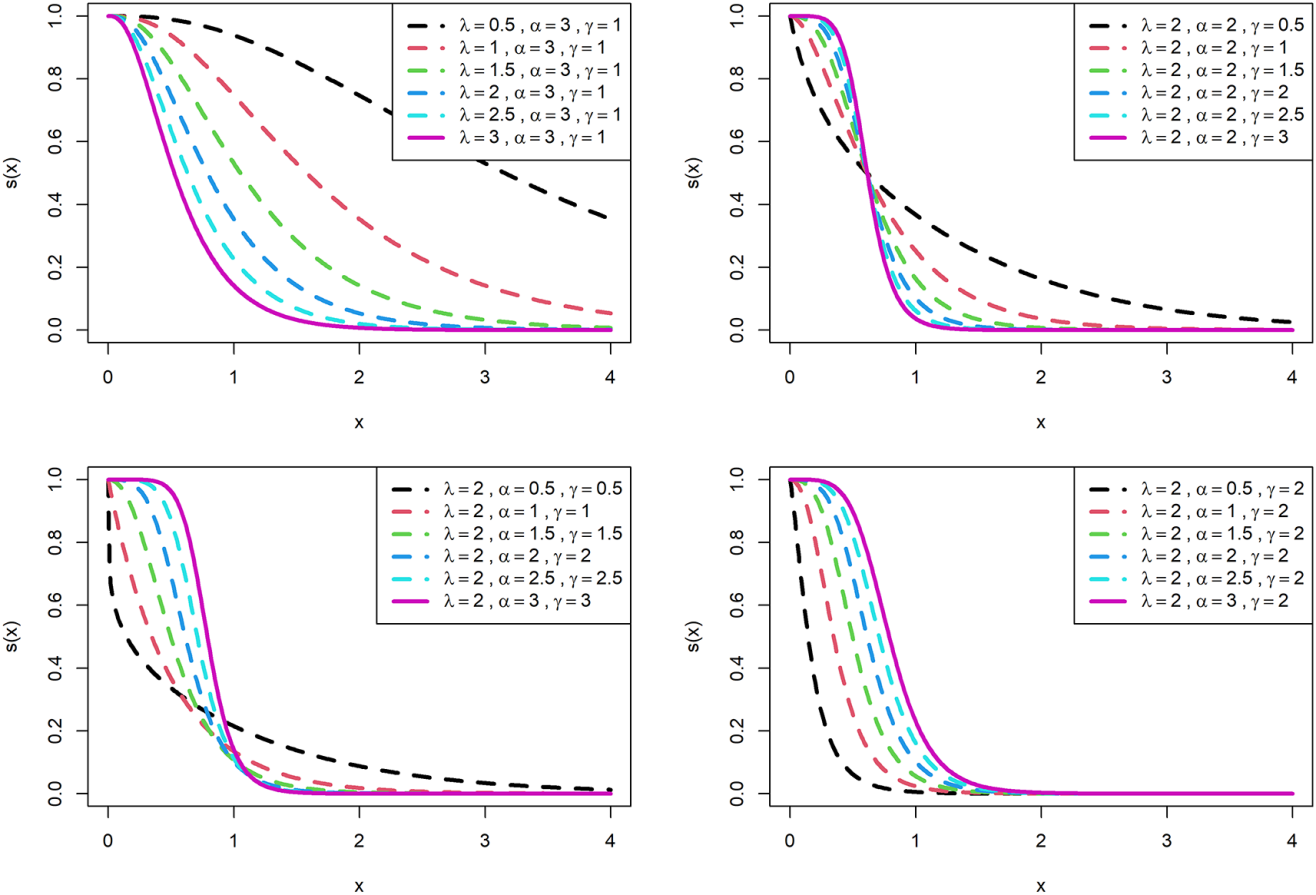
Visual presentation of survival function plots of the OLLGED for various parameters.

**Figure 4.**
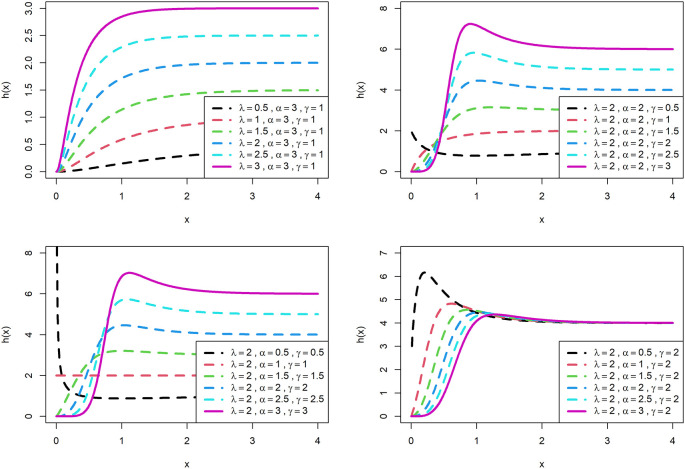
Visual presentation of hazard rate plots of the OLLGED for various parameters.

The visualization of survival functions and hazard rates of OLLGED for various parametric values are presented in Figures 3 and 4. Supplementary figures 3 and 4 disclose that this family can generate $h\left(x\right)$ shapes for instance increasing, reversed-J, decreasing, constant, and upside-down bathtubs. This shows that the OLLGE family could be extremely practical to fit data sets for diversified shapes.

## 3. Properties

### 3.1 Useful expansions

Using Taylor’s series specifically binomial series expansion for expansion of CDF and PDF for distribution as derived by OLLGED enables us to obtain the following functions as alternatives to the Equations given as PDF and CDF in equation (5) and (6) respectively. At this juncture, the CDF of OLLGED can be written using binomial expansion of its expressions as it was derived in Ref. 20 while expressing in much more simplified form parts of the CDF equations see in equation (9) and then substituted in the equation (6) to obtain the CDF see equation (10):

$${\left[1-e^{-\mathrm{\lambda x}}\right]}^{\alpha \gamma }=\sum_{k=0}^{\infty }a_{k}{\left[1-e^{-\mathrm{\lambda x}}\right]}^{k}.$$ (9)

Whereas, $a_{k}=a_{k}\left(\alpha \gamma \right)=\sum_{j=k}^{\infty }{\left(-1\right)}^{k+1}\left(\begin{array}{c} \alpha \gamma \\ j \end{array}\right)\left(\begin{array}{c} j\\ k \end{array}\right)$ .

The generalized binomial expansion is considered for γ>0 :

$${\left[1-{\left(1-e^{-\mathrm{\lambda x}}\right)}^{\alpha }\right]}^{\gamma }=\sum_{k=0}^{\infty }a_{k}^{*}{\left[1-e^{-\mathrm{\lambda x}}\right]}^{k}\;$$ (10)

Where $a_{k}^{*}=\sum_{i=0}^{\infty }\sum_{j=k}^{\infty }{\left(-1\right)}^{i+k+j}\left(\begin{array}{c} \gamma \\ i \end{array}\right)\left(\begin{array}{c} \alpha \\ j \end{array}\right)\left(\begin{array}{c} j\\ k \end{array}\right)$ .

Thus, the CDFs of the OLLGED can be expressed as follows:

$$G\left(x\right)=\frac{\sum_{k=0}^{\infty }a_{k}{\left[1-e^{-\mathrm{\lambda x}}\right]}^{k}}{\sum_{k=0}^{\infty }b_{k}{\left[1-e^{-\mathrm{\lambda x}}\right]}^{k}}.$$ (11)

Where $b_{k}=a_{k}+a_{k}^{*}$ .

The following expression is for the ratio of the two-power series:

$$G\left(x\right)=\sum_{k=0}^{\infty }c_{k}{\left[1-e^{-\mathrm{\lambda x}}\right]}^{k}\;$$ (12)

Where $c_{0}=\frac{a_{0}}{b_{0}}$ and the coefficients of CK for k≥1 are determined from the recurrence generator which is given as:

$$c_{k}=b_{0}^{-1}\left(a_{k}-b_{0}^{-1}\sum_{r=0}^{k}b_{r}c_{k-r}\right).$$ (13)

Thus, PDF becomes

$$g\left(x\right)=\sum_{k=0}^{\infty }c_{k+1}\lambda e^{-\mathrm{\lambda x}}\left(k+1\right){\left(1-e^{-\mathrm{\lambda x}}\right)}^{k}.$$ (14)

### 3.2 Quantile function

The quantile function of the OLLGED is given by derivations while considering important theories.

Recalling the function for the quantile of the probability distribution to be given as:

$$G\left(X<x\right)=q$$

Insert equation (10) in equation (18), and solve for the variable *x* we get

$$x_{q}=\frac{-1}{\lambda }\ln\left[1-{\left\{1+{\left(q^{-1}-1\right)}^{\frac{1}{\gamma }}\right\}}^{\frac{-1}{\alpha }}\right].$$ (15)

Upon substituting the appropriate value of quantile q , we will be able to obtain its quantile value $\left(x_{q}\right)$ .

### 3.3 Moments and generating functions

The $r^{\mathrm{th}}$ moment for the OLLGED is given as:

$$\mu_{r}^{\prime }=E\left(x^{r}\right)=\int_{0}^{\infty }x^{r}g\left(x\right)\mathrm{dx}.$$ (16)

Since $g\left(x\right)=\sum_{k=0}^{\infty }c_{k+1}\lambda e^{-\mathrm{\lambda x}}\left(k+1\right){\left(1-e^{-\mathrm{\lambda x}}\right)}^{k}$

Thus, we get

$$\begin{array}{c} \mu_{r}^{\prime }=\int_{0}^{\infty }x^{r}\sum_{k=0}^{\infty }c_{k+1}\lambda e^{-\mathrm{\lambda x}}\left(k+1\right){\left(1-e^{-\mathrm{\lambda x}}\right)}^{k}\mathrm{dx}\\ \;=\sum_{k=0}^{\infty }\left(k+1\right)\lambda c_{k+1}\int_{0}^{\infty }x^{r}e^{-\mathrm{\lambda x}}{\left(1-e^{-\mathrm{\lambda x}}\right)}^{k}\mathrm{dx} \end{array}$$ (17)

Now consider ${\left(1-e^{-\mathrm{\lambda x}}\right)}^{k}=\sum_{j=0}^{\infty }{\left(-1\right)}^{j}\left(\begin{array}{c} k\\ j \end{array}\right)e^{-\mathrm{\lambda kx}}$ .

Then we obtain equation (17) as follows:

$$\begin{array}{c} \mu_{r}^{\prime }=\sum_{k=0}^{\infty }\left(k+1\right)\lambda c_{k+1}\int_{0}^{\infty }x^{r}e^{-\mathrm{\lambda x}}\sum_{j=0}^{\infty }{\left(-1\right)}^{j}\left(\begin{array}{c} k\\ j \end{array}\right)e^{-\mathrm{\lambda kx}}\mathrm{dx}\\ \;=\sum_{k=0}^{\infty }\sum_{j=0}^{\infty }{\left(-1\right)}^{j}\left(\begin{array}{c} k\\ j \end{array}\right)\left(k+1\right)\lambda c_{k+1}\int_{0}^{\infty }x^{r}e^{-\mathrm{\lambda x}\left(1+k\right)}\mathrm{dx}\\ \;=\lambda \sum_{k=0}^{\infty }\sum_{j=k}^{\infty }{\left(-1\right)}^{j}\left(\begin{array}{c} k\\ j \end{array}\right)\left(k+1\right)c_{k+1}\frac{\Gamma \left(r+1\right)}{{\left[\left(1+k\right)\lambda \right]}^{\left(r+1\right)}}. \end{array}$$ (18)

Where $\int_{0}^{\infty }x^{r}e^{-\mathrm{\lambda x}\left(1+k\right)}\mathrm{dx}=\frac{\Gamma \left(r+1\right)}{{\left[\left(1+k\right)\lambda \right]}^{\left(r+1\right)}}$

Therefore, mean is given by:

$$\mathrm{Mean}=\mu_{1}^{\prime }=\frac{1}{\lambda }\sum_{k=0}^{\infty }\sum_{j=k}^{\infty }{\left(-1\right)}^{j}\left(\begin{array}{c} k\\ j \end{array}\right)\left(k+1\right)c_{k+1}\frac{1}{{\left[\left(1+k\right)\right]}^{2}}.$$ (19)

$$\mu_{2}^{\prime }=\frac{1}{\lambda^{2}}\sum_{k=0}^{\infty }\sum_{j=0}^{\infty }{\left(-1\right)}^{j}\left(\begin{array}{c} k\\ j \end{array}\right)\left(k+1\right)c_{k+1}\frac{2}{{\left(1+k\right)}^{3}}$$

$$\begin{array}{c} \text{Variance}\;\mu_{2}=\mu_{2}^{\prime }-{\left(\mu_{1}^{\prime }\right)}^{2}\\ =\frac{1}{\lambda^{2}}\left\{2\sum_{k=0}^{\infty }\sum_{j=k}^{\infty }{\left(-1\right)}^{j}\left(\begin{array}{c} k\\ j \end{array}\right)\left(k+1\right)c_{k+1}\frac{1}{{\left(1+k\right)}^{3}}-{\left(\sum_{k=0}^{\infty }\sum_{j=k}^{\infty }{\left(-1\right)}^{j}\left(\begin{array}{c} k\\ j \end{array}\right)\left(k+1\right)c_{k+1}\frac{1}{{\left(1+k\right)}^{2}}\right)}^{2}\right\} \end{array}$$ (20)

Moment generating function for the OLLGED is derived in the following manner:

$$\begin{array}{l} M_{x}\left(t\right)=E\left(e^{\mathrm{tx}}\right)\\ \;=\int_{0}^{\infty }e^{\mathrm{tx}}\sum_{k=0}^{\infty }c_{k+1}\lambda e^{-\mathrm{\lambda x}}\left(k+1\right){\left(1-e^{-\mathrm{\lambda x}}\right)}^{k}\mathrm{dx}\\ \;=\lambda \sum_{k=0}^{\infty }c_{k+1}\left(k+1\right)\int_{0}^{\infty }e^{\mathrm{tx}}e^{-\mathrm{\lambda x}}{\left(1-e^{-\mathrm{\lambda x}}\right)}^{k}\mathrm{dx}\\ \;=\lambda \sum_{k=0}^{\infty }\sum_{j=k}^{\infty }{\left(-1\right)}^{j}\left(\begin{array}{c} k\\ j \end{array}\right)c_{k+1}\left(k+1\right)\int_{0}^{\infty }e^{\mathrm{tx}}e^{-\mathrm{\lambda x}}e^{-\mathrm{\lambda kx}}\mathrm{dx}\\ \;=\lambda \sum_{k=0}^{\infty }\sum_{j=0}^{\infty }{\left(-1\right)}^{j}\left(\begin{array}{c} k\\ j \end{array}\right)c_{k+1}\left(k+1\right)\frac{1}{\left(t-\lambda -\mathrm{\lambda k}\right)}. \end{array}$$ (21)

Where ${\left(1-e^{-\mathrm{\lambda x}}\right)}^{k}=\sum_{j=0}^{\infty }{\left(-1\right)}^{j}\left(\begin{array}{c} k\\ j \end{array}\right)e^{-\mathrm{\lambda kx}}$ .

### 3.4 Skewness and Kurtosis

Since the moment cannot be obtained easily, in such a case, there are several methods for evaluating Skewness and Kurtosis in literature. Some of the famous methods are Galton's Skewness $\left(S_{k}\right)$ and Moor’s Kurtosis $\left(M_{k}\right)$ methods, ^26^ both of which utilize octile of the distribution.

Galton skewness of the distribution is given by considering octiles as follows:

$$S_{k}=\frac{Q\left(\frac{6}{8}\right)+Q\left(\frac{2}{8}\right)-2Q\left(\frac{4}{8}\right)}{Q\left(\frac{6}{8}\right)-Q\left(\frac{2}{8}\right)}.$$ (22)

**Figure 5.**
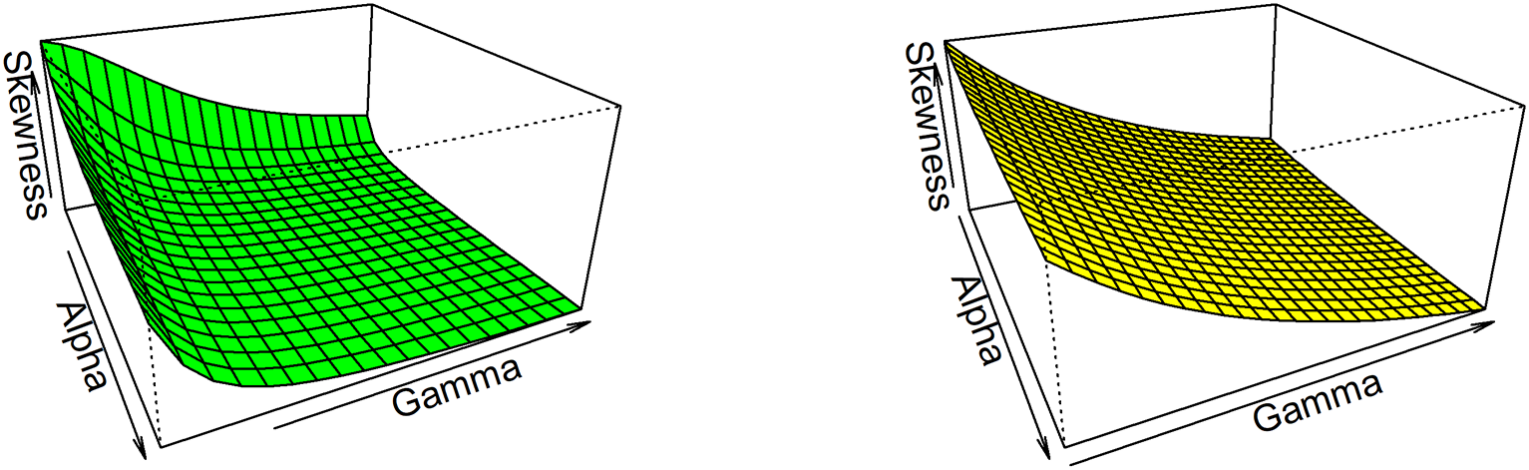
Visual presentation for Skewness of the OLLGED.

Thus, based on varying values of distributional parameters, various values of skewness can be obtained and Figure 5 displayed the 3-dimensional plot of the skewness of the distribution. From Figure 5 it is evident that the skewness decreases as both γandα increase when λ=1 .

While for kurtosis, Moor’s Kurtosis $\left(M_{k}\right)$ method is used, which is based on octiles and it is given by:

$$M_{k}=\frac{Q\left(\frac{7}{8}\right)-Q\left(\frac{5}{8}\right)+Q\left(\frac{3}{8}\right)-Q\left(\frac{1}{8}\right)}{Q\left(\frac{6}{8}\right)-Q\left(\frac{2}{8}\right)}.$$ (23)

**Figure 6.**
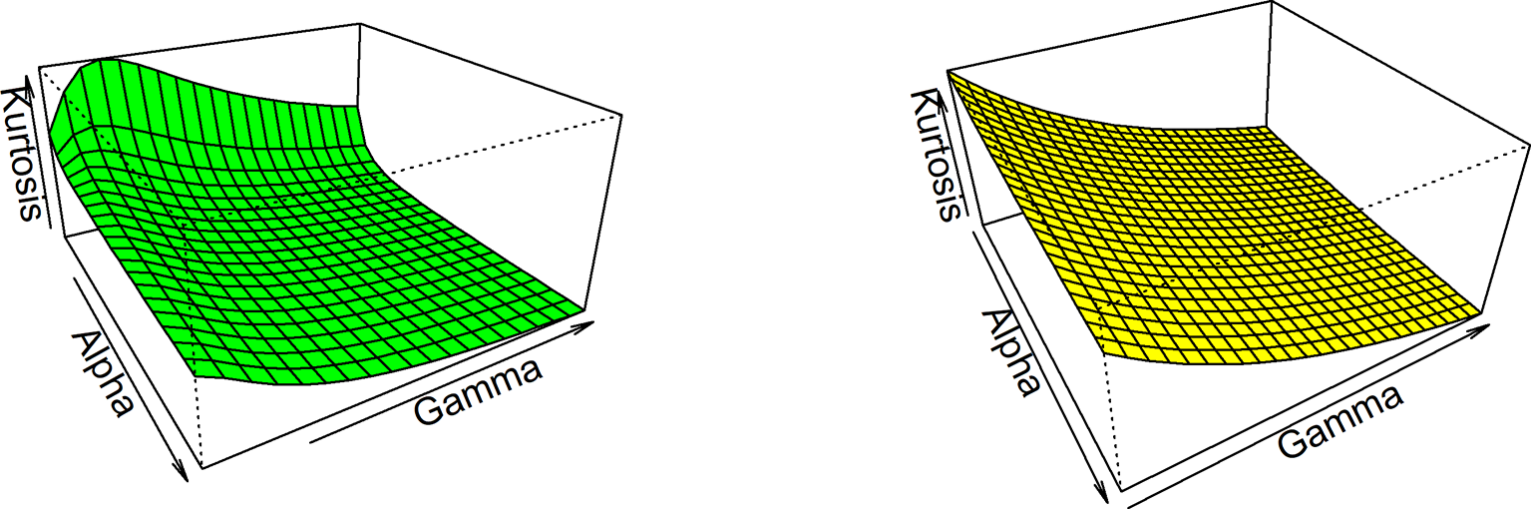
The Kurtosis of the OLLGED.

A 3-dimensional plot for varying values of distributional parameters is presented in Figure 6. From Figure 5 it is clear that the kurtosis decreases as both γandα increase when λ=1 . The moments, skewness, and kurtosis for various parametric combinations are given in Table 1. When we fix the parameter λ, the skewness and kurtosis of OLLGED increases as *α* and γ increases. More specifically when parametric values are increases the skewness becomes negative and kurtosis becomes mesokurtic.

**Table 1.** The calculated moments, skewness, and kurtosis measures of the OLLGED for selected parameter values.

λ	α	γ	Mean	Variance	Skewness	Kurtosis
1.0	1.0	1.0	1.0000	1.0000	0.1918	1.2062
1.0	2.0	2.0	1.3079	0.3066	-0.0054	0.9772
1.1	1.1	1.1	0.9260	0.7008	0.1503	1.1137
1.2	1.2	1.2	0.8694	0.5073	0.1173	1.0327
1.3	1.3	1.3	0.8248	0.3772	0.0908	0.9619
1.4	1.4	1.4	0.7884	0.2868	0.0691	0.9001
1.5	1.5	1.5	0.7579	0.2223	0.0512	0.8460
1.6	1.6	1.6	0.7318	0.1752	0.0361	0.7985
1.6	2.6	2.6	0.9368	0.0760	-0.0387	0.7068
1.7	1.7	1.7	0.7090	0.1400	0.0233	0.7567
1.7	2.7	2.7	0.8991	0.0630	-0.0425	0.6767
1.8	1.8	1.8	0.6888	0.1133	0.0124	0.7196
1.8	2.8	2.8	0.8651	0.0527	-0.0459	0.6495
1.9	1.9	1.9	0.6705	0.0927	0.0029	0.6866
1.9	2.9	2.9	0.8344	0.0444	-0.0491	0.6249
2.0	2.0	2.0	0.6540	0.0766	-0.0054	0.6571
2.1	2.1	2.1	0.6387	0.0639	-0.0127	0.6306
2.2	2.2	2.2	0.6246	0.0538	-0.0191	0.6068
2.3	2.3	2.3	0.6114	0.0455	-0.0248	0.5852
2.4	2.4	2.4	0.5991	0.0388	-0.0299	0.5655
2.5	2.5	2.5	0.5875	0.0333	-0.0345	0.5476
2.6	2.6	2.6	0.5765	0.0288	-0.0387	0.5313
2.7	2.7	2.7	0.5661	0.0250	-0.0425	0.5163
2.8	2.8	2.8	0.5562	0.0218	-0.0459	0.5024
2.9	2.9	2.9	0.5467	0.0191	-0.0491	0.4897
3.0	3.0	3.0	0.5376	0.0168	-0.0520	0.4779
3.1	3.1	3.1	0.5290	0.0148	-0.0546	0.4669
3.2	3.2	3.2	0.5207	0.0131	-0.0571	0.4567
3.3	3.3	3.3	0.4467	0.0043	-0.0594	0.4472
3.4	3.4	3.4	0.5050	0.0104	-0.0615	0.4384
3.5	3.5	3.5	0.4976	0.0093	-0.0634	0.4301
3.6	3.6	3.6	0.4905	0.0084	-0.0653	0.4223
3.7	3.7	3.7	0.4836	0.0076	-0.0670	0.4150
3.8	3.8	3.8	0.4769	0.0068	-0.0686	0.4081
3.9	3.9	3.9	0.4705	0.0062	-0.0701	0.4016
4.0	4.0	4.0	0.4643	0.0056	-0.0715	0.3955
4.1	4.1	4.1	0.4582	0.0051	-0.0728	0.3897
4.2	4.2	4.2	0.4524	0.0047	-0.0741	0.3843
4.3	4.3	4.3	0.4467	0.0043	-0.0753	0.3791
4.4	4.4	4.4	0.4412	0.0039	-0.0764	0.3741
4.5	3.5	3.5	0.3870	0.0057	-0.0634	0.3798
4.5	4.5	4.5	0.4359	0.0036	-0.0775	0.3695
4.6	3.6	3.6	0.3839	0.0051	-0.0653	0.3746
4.6	4.6	4.6	0.4307	0.0033	-0.0785	0.3650
4.7	4.7	4.7	0.4256	0.0030	-0.0794	0.3608
4.8	4.8	4.8	0.4207	0.0028	-0.0804	0.3567
4.9	4.9	4.9	0.4159	0.0026	-0.0812	0.3529
5.0	5.0	5.0	0.4113	0.0024	-0.0821	0.3492

### 3.5 Residual and reversed residual life

For the residual life, $n^{\mathrm{th}}$ moment is generally given as, $m_{n}\left(t\right)=E\left[\left.{\left(X-t\right)}^{t}\right|X>t\right]$ , n=1,2,3,4,… which is uniquely determined for the cumulative function $F\left(x\right)$ . Assuming *X* to be a random lifetime variable with $F\left(x\right)$ then the residual life $n^{\mathrm{th}}$ moment is obtained as $m_{n}\left(t\right)=\frac{1}{R\left(t\right)}\int_{t}^{\infty }{\left(X-t\right)}^{n}\mathrm{dF}\left(x\right)$ .

Many other functions are derived from the residual life $n^{\mathrm{th}}$ moment such as mean residual life (MRLF) or life expectation at time *t* defined by:

$m_{1}\left(t\right)=E\left[\left.\left(X-t\right)\right|X>t\right]$ , this presents the expected additional life length for a unit that is alive at time *t*.

The reversed residual life $n^{\mathrm{th}}$ moment is generally defined as, $m_{n}\left(t\right)=E\left[\left.{\left(X-t\right)}^{t}\right|X\leq t\right]$ only defined for t>0 and n=1,2,3,4,… , then, can be used to determine uniquely $F\left(x\right)$ .

Thus, the mean inactivity time (MIT) also referred to as mean waiting time (MWT) or mean reversed residual lifetime given by; $m_{1}\left(t\right)=E\left[\left.\left(X-t\right)\right|X\leq t\right]$ , which is the waiting time, since the failure of an item on condition that the failure has occurred in (0, *t*).

### 3.6 Order statistics

In practice, most of the events occur randomly following a chronological order either ascending or descending. Thus, their probability distribution properties such as CDF and PDF can be written taking into consideration such criteria of their orders. The order statistics consider the order of occurrence of a random variable. Suppose that *X*_1_, *X*_2_ … *X*_*n*_, is a random sample from the OLLGED, in the ascending values of the ordered random variables as $X_{1;n}\leq X_{2;n}\leq ,\ldots ,\leq X_{n;n}$ , the PDF of the *j* ^th^ order statistic, say *X*_*j*; *n*_, is given in the next equation (24):

$$f_{j;n}\left(x\right)=\frac{g\left(x\right)}{B\left(j,n-j+1\right)}\sum_{i=0}^{n-j}{\left(-1\right)}^{i}\left(\begin{array}{c} n-j\\ i \end{array}\right)G^{i+j-1}\left(x\right)\;$$ (24)

Whereas, $B\left(j,n-j+1\right)$ is the beta function.

Upon substitution of equations (9) and (10) in equation (24) we get the following expression:

$$f_{j;n}\left(x\right)=\sum_{i=o}^{n-j}\sum_{r,k=0}^{\infty }m_{i,r,k}h_{r+k+1}\left(x\right)$$

Where $h_{r+k+1}\left(x\right)$ denotes the probability density function for OLLGED having *r*+ *k*+1 power parameter.

$$m_{i,r,k}=\frac{{\left(-1\right)}^{i}\left(r+1\right)c_{r+1}f_{i+j-1,k}}{B\left(j,n-j+1\right)\left(r+k+1\right)}.$$

Where, $c_{k}=b_{0}^{-1}\left(a_{k}-b_{0}^{-1}\sum_{r=0}^{k}b_{r}c_{k-r}\right)$ , hence the quantity $f_{i+j-1,k}$ is obtained recursively by $f_{i+j-1,0}=c_{0}^{i+j-1}$ and for values of k≥1 .

$$f_{i+j-1,k}={\left(kc_{0}\right)}^{-1}\sum_{m=1}^{k}\left[m\left(i+j\right)-k\right]c_{m}f_{i+j-1},k-m.$$

Therefore, the density function of the OLLGED order statistics is a combination of GED. Based on $f_{i+j-1,k}$ , it is noted that the properties of $X_{i;n}$ follow from the properties of $X_{r+k+1}$ . Thus, the moment of $X_{i;n}$ can be expressed as:

$$E\left(X_{i;n}^{q}\right)=\sum_{j=0}^{n-i}\sum_{r,k=0}^{\infty }m_{j,r,k}E\left(X_{r+k+1}^{q}\right)$$ (25)

Consider moment in equation (25) for the derivation of explicit expression for L-moments of X as infinite weighted linear combinations of suitable OLLGED order statistics defined as a linear function as:

$$\lambda_{r}=\frac{1}{r}\sum_{d=0}^{r-1}{\left(-1\right)}^{d}\left(\begin{array}{c} r-1\\ d \end{array}\right)E\left(X_{r-d:r}\right),r\geq 1.$$

## 4. Parametric estimation

The consideration of the unknown OLLGED model parameters from the complete samples is determined by using maximum likelihood estimations (MLE) as it is commonly used in the literature, ^27^ which for OLLGED parameters are λ,α,andγ . Assuming $x_{1},x_{2},\ldots ,x_{n}$ be a random sample from OLLGED, the log-likelihood function is given by:

$$\log L=\text{nlog}\gamma +\sum_{i=0}^{n}\log g\left(x\right)+\left(\gamma -1\right)\sum_{i=0}^{n}\log G\left(x\right)+\left(\gamma -1\right)\sum_{i=0}^{n}\log\bar{G}\left(x\right)-2\left(\gamma -1\right)\sum_{i=0}^{n}\log\left\{G^{\gamma }\left(x\right)+{\bar{G}}^{\gamma }\left(x\right)\right\}.$$

Upon finding the second derivative, we obtain the following equations:

∂2logL∂λ2=∑i=1ngλλxigxi−gλxi2g2xi+γ−1∑i=1nGλλxiGxi−Gλxi2Gxi2+γ−1∑i=1nGλxi2−GλλxiG¯xiG¯2xi−2γ∑i=1nγ−1GλλxiGλxiGxiγ−2−GλλxiG¯xiγ−2GλxiGxiγ+G¯xiγ−γGλxiGxiγ−1−G¯xiγ−1GλxiGλxiGxiγ−1−GλxiG¯xiγ−1.

Similarly, second derivatives concerning parameters are obtained $\left(\frac{\partial^{2}\log L}{\partial \lambda \partial \alpha },\frac{\partial^{2}\log L}{\partial \alpha^{2}},\frac{\partial^{2}\log L}{\partial \lambda \partial \gamma },\frac{\partial^{2}\log L}{\partial \gamma^{2}}\right.$ and $\left.\frac{\partial^{2}\log L}{\partial \alpha \partial \gamma }\right)$

hence an information matrix is formed and given as:

$$I=\left[\begin{array}{ccc} \frac{\partial^{2}\log L}{\partial \lambda^{2}} & \frac{\partial^{2}\log L}{\partial \lambda \partial \alpha } & \frac{\partial^{2}\log L}{\partial \lambda \partial \gamma }\\ \frac{\partial^{2}\log L}{\partial \lambda \partial \alpha } & \frac{\partial^{2}\log L}{\partial \alpha^{2}} & \frac{\partial^{2}\log L}{\partial \alpha \partial \gamma }\\ \frac{\partial^{2}\log L}{\partial \lambda \partial \gamma } & \frac{\partial^{2}\log L}{\partial \alpha \partial \gamma } & \frac{\partial^{2}\log L}{\partial \gamma^{2}} \end{array}\right].$$

Since it seems not possible to solve the obtained MLE of parametric estimates analytically, then it is wise to solve these estimates using softwares such as R (an open source software for statistical computing and graphics) and SAS (an integrated software suite for advanced analytics, business intelligence, data management, and predictive analytics), we can find MLE for the OLLGED parameters or else find the solution to obtained non-linear likelihood equations. For the sake of this research work, the analysis is carried out using the R statistical software ^28^ to obtain parametric values for the MLE estimate of the suggested OLLGED.

## 5. Simulation study

This section deals with the behavior of the MLEs of the unknown parameters of the proposed OLLGED has been assessed through simulation. The simulation study is carried out for sample sizes *n* = 50, 100, 150, 200, 250, and 300 from OLLGED with 6 combinations of parameters. To evaluate the performance of the MLEs for the OLLGED model, the simulation study was performed as follows: Generate *B* = 3000 samples of size *n* from $\text{OLLGED}\left(\lambda ,\alpha ,\gamma \right)$ , compute the MLE for the *B* samples, say $\left({\hat{\lambda }}_{j},{\hat{\alpha }}_{j},{\hat{\gamma }}_{j}\right);j=1,2,\ldots ,B$ . Compute the biases and mean squared errors (MSE) based on *B* samples. We repeated these steps for *n* = 50, 100, 150, 200, 250, and 300 with different values of $\;\left(\lambda ,\alpha ,\gamma \right)$ . To estimate the MLEs, the Broyden–Fletcher–Goldfarb–Shanno (BFGS) method in R software was used. Table 2 gives empirical results and its values reveal that the estimates are quite stable and, meaningfully, are near to the actual value of the parameters as the sample size increases for all parameters. The bias and mean square error (MSE) of both parameters decrease as the sample size increases as anticipated. The bias and MSE of the parameters are obtained as follows:

$$\text{bias}=\frac{1}{B}\sum_{i=1}^{B}\left({\hat{\theta }}_{i}-\theta \right)$$

**Table 2.** Average bias and MSE of OLLGED for various parametric combinations.

n	True values			Bias			MSE		
	λ	α	γ	λ	α	γ	λ	α	γ
50	1.5	1.5	0.2	0.2110	0.2793	0.0426	0.7762	0.9875	0.0311
100	1.5	1.5	0.2	0.1046	0.1317	0.0253	0.4006	0.4589	0.0142
150	1.5	1.5	0.2	0.0507	0.0682	0.0188	0.2507	0.2905	0.0083
200	1.5	1.5	0.2	0.0377	0.0491	0.0136	0.1839	0.2108	0.0055
250	1.5	1.5	0.2	0.0193	0.0269	0.0124	0.1452	0.1680	0.0042
300	1.5	1.5	0.2	0.0176	0.0230	0.0099	0.1168	0.1344	0.0033
50	0.2	0.2	1.5	0.4922	0.1680	-0.0593	2.2882	0.3726	0.4528
100	0.2	0.2	1.5	0.1647	0.0471	-0.0175	0.3980	0.0527	0.2235
150	0.2	0.2	1.5	0.0783	0.0199	-0.0117	0.0798	0.0102	0.1299
200	0.2	0.2	1.5	0.0511	0.0121	-0.0076	0.0285	0.0024	0.0916
250	0.2	0.2	1.5	0.0386	0.0093	-0.0061	0.0192	0.0017	0.0715
300	0.2	0.2	1.5	0.0313	0.0077	-0.0039	0.0150	0.0015	0.0604
50	0.2	0.2	1.0	0.2612	0.1174	-0.0375	0.6483	0.1617	0.1540
100	0.2	0.2	1.0	0.0826	0.0318	-0.0128	0.0861	0.0187	0.0688
150	0.2	0.2	1.0	0.0443	0.0168	-0.0070	0.0274	0.0060	0.0419
200	0.2	0.2	1.0	0.0295	0.0108	-0.0056	0.0115	0.0018	0.0292
250	0.2	0.2	1.0	0.0219	0.0082	-0.0027	0.0080	0.0013	0.0231
300	0.2	0.2	1.0	0.0186	0.0073	-0.0025	0.0065	0.0011	0.0194
50	1.0	0.2	1.0	1.2950	0.1185	-0.0353	16.0392	0.1693	0.1545
100	1.0	0.2	1.0	0.4037	0.0315	-0.0112	2.1329	0.0188	0.0685
150	1.0	0.2	1.0	0.2139	0.0164	-0.0054	0.6860	0.0062	0.0414
200	1.0	0.2	1.0	0.1409	0.0104	-0.0041	0.2811	0.0018	0.0289
250	1.0	0.2	1.0	0.1028	0.0078	-0.0009	0.1964	0.0013	0.0229
300	1.0	0.2	1.0	0.0871	0.0069	-0.0008	0.1578	0.0011	0.0193
50	0.2	1.5	0.2	0.0285	0.2780	0.0432	0.0139	0.9841	0.0313
100	0.2	1.5	0.2	0.0125	0.1184	0.0288	0.0073	0.4649	0.0154
150	0.2	1.5	0.2	0.0051	0.0530	0.0223	0.0047	0.3006	0.0092
200	0.2	1.5	0.2	0.0030	0.0309	0.0174	0.0035	0.2204	0.0063
250	0.2	1.5	0.2	0.0001	0.0058	0.0167	0.0029	0.1798	0.0050
300	0.2	1.5	0.2	-0.0002	0.0015	0.0140	0.0023	0.1444	0.0040
50	1.0	1.5	0.2	0.1403	0.2767	0.0424	0.3440	0.9796	0.0309
100	1.0	1.5	0.2	0.0674	0.1285	0.0258	0.1771	0.4563	0.0144
150	1.0	1.5	0.2	0.0330	0.0668	0.0191	0.1112	0.2903	0.0084
200	1.0	1.5	0.2	0.0242	0.0457	0.0139	0.0820	0.2075	0.0055
250	1.0	1.5	0.2	0.0129	0.0261	0.0126	0.0652	0.1679	0.0043
300	1.0	1.5	0.2	0.0115	0.0222	0.0099	0.0512	0.1313	0.0033

$\mathrm{MSE}=\frac{1}{B}\sum_{i=1}^{B}{\left({\hat{\theta }}_{i}-\theta \right)}^{2}$ . Where $\theta =\left(\lambda ,\alpha ,\gamma \right)$ .

## 6. Data analysis

The following two data sets were used to reveal the applications of OLLGED for showing the flexibility and importance of the proposed distribution. For the application of the OLLGED using the first data set for illustration, the data represent waiting times (in seconds) between 65 successive eruptions of water through a hole in the cliff at the coastal town of Kiama (New South Wales, Australia), known as the Blowhole; the data can be obtained from http://www.statsci.org/data/oz/kiama.html. This data set has already been used ^29^: DOI: http://dx.doi.org/10.15446/rce.v42n1.66205 as follows: 83, 51, 87, 60, 28, 95, 8, 27, 15, 10, 18, 16, 29, 54, 91, 8, 17, 55, 10, 35,47, 77, 36, 17, 21, 36, 18, 40, 10, 7, 34, 27, 28, 56, 8, 25, 68, 146, 89, 18, 73, 69, 9, 37, 10, 82, 29, 8, 60, 61, 61, 18, 169, 25, 8, 26, 11, 83, 11, 42, 17, 14, 9, 12.

The second data set used here was the survival times (given in years) of a group comprising 46 patients treated with chemotherapy alone. This data set was earlier reported ^18^^,^^30^; doi: https://doi.org/10.1016/j.joems.2014.12.002, for ready reference, the survival times (years) are 0.047, 0.115, 0.121, 0.132, 0.164, 0.197, 0.203, 0.260, 0.282, 0.296, 0.334, 0.395, 0.458, 0.466, 0.501, 0.507,0.529,0.534,0.540, 0.641, 0.644, 0.696, 0.841, 0.863, 1.099, 1.219, 1.271, 1.326, 1.447, 1.485, 1.553, 1.581, 1.589, 2.178, 2.343, 2.416, 2.444, 2.825, 2.830, 3.578, 3.658, 3.743, 3.978, 4.003, 4.033.

Furthermore, the developed OLLGED fits were compared with other models like odd generalized exponential log-logistic distribution (OGELLD), ^31^ Type-II generalized log-logistic distribution (ELLD), ^32^ odd exponential log-logistic distribution (OELLD), ^33^ generalized exponential distribution (GED), ^6^ exponential distribution (ED) and log-logistic distribution (LLD) studied by. ^25^^,^^34^ The competency of the proposed model with other models is examined based on goodness-of-fit criteria such as the maximized log-likelihood under the model ( $-2\hat{l}$ ), Akaike information criterion (AIC), Bayesian information criterion (BIC), Anderson-Darling (A ^*^), Cramer-von Mises (W ^*^) and Kolmogorov Smirnov (KS) statistic along with its *p*-value.

**Figure 7.**
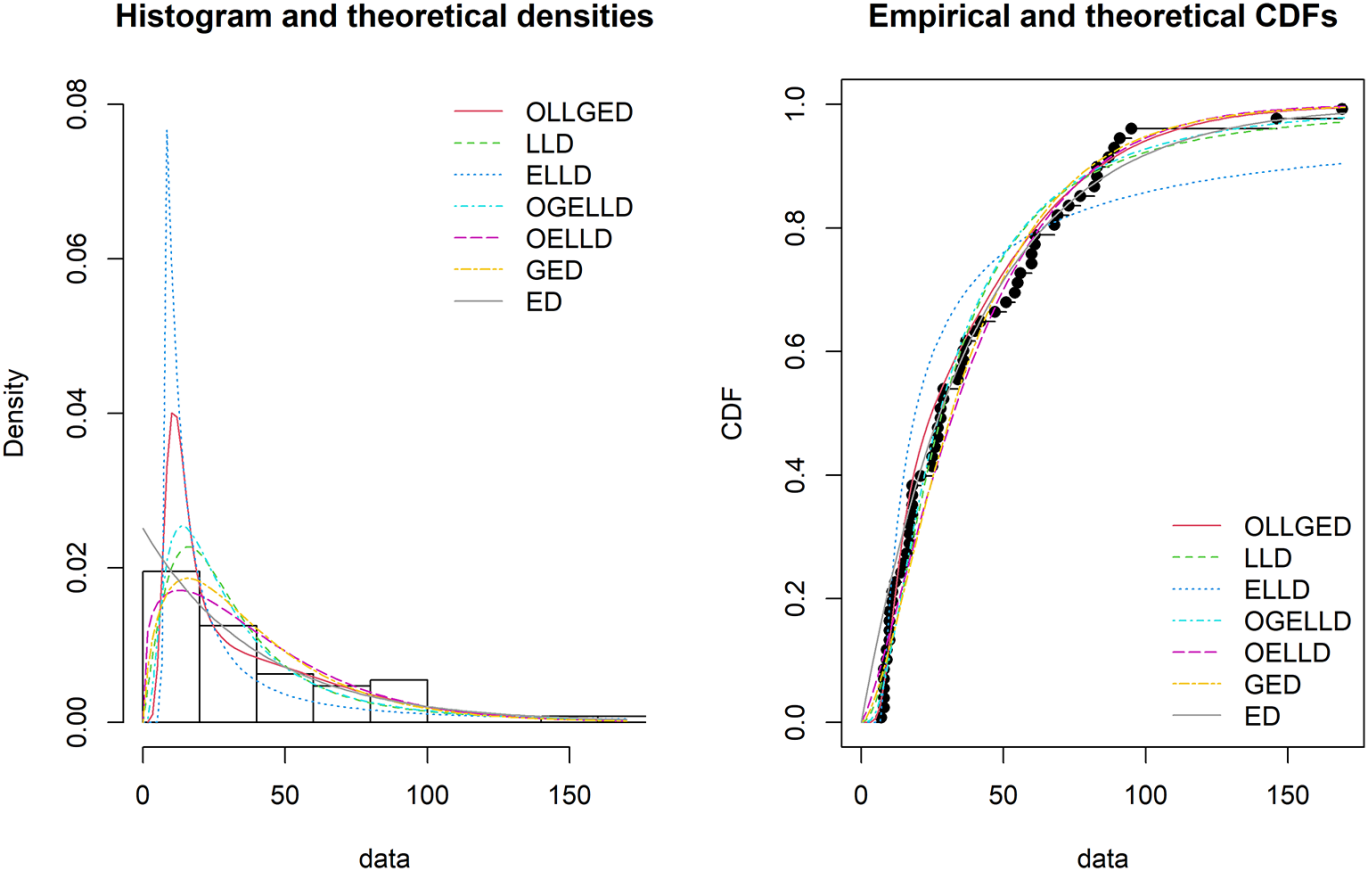
The densities fitted (left) and CDF plots (right) for various models for waiting time data.

**Figure 8.**
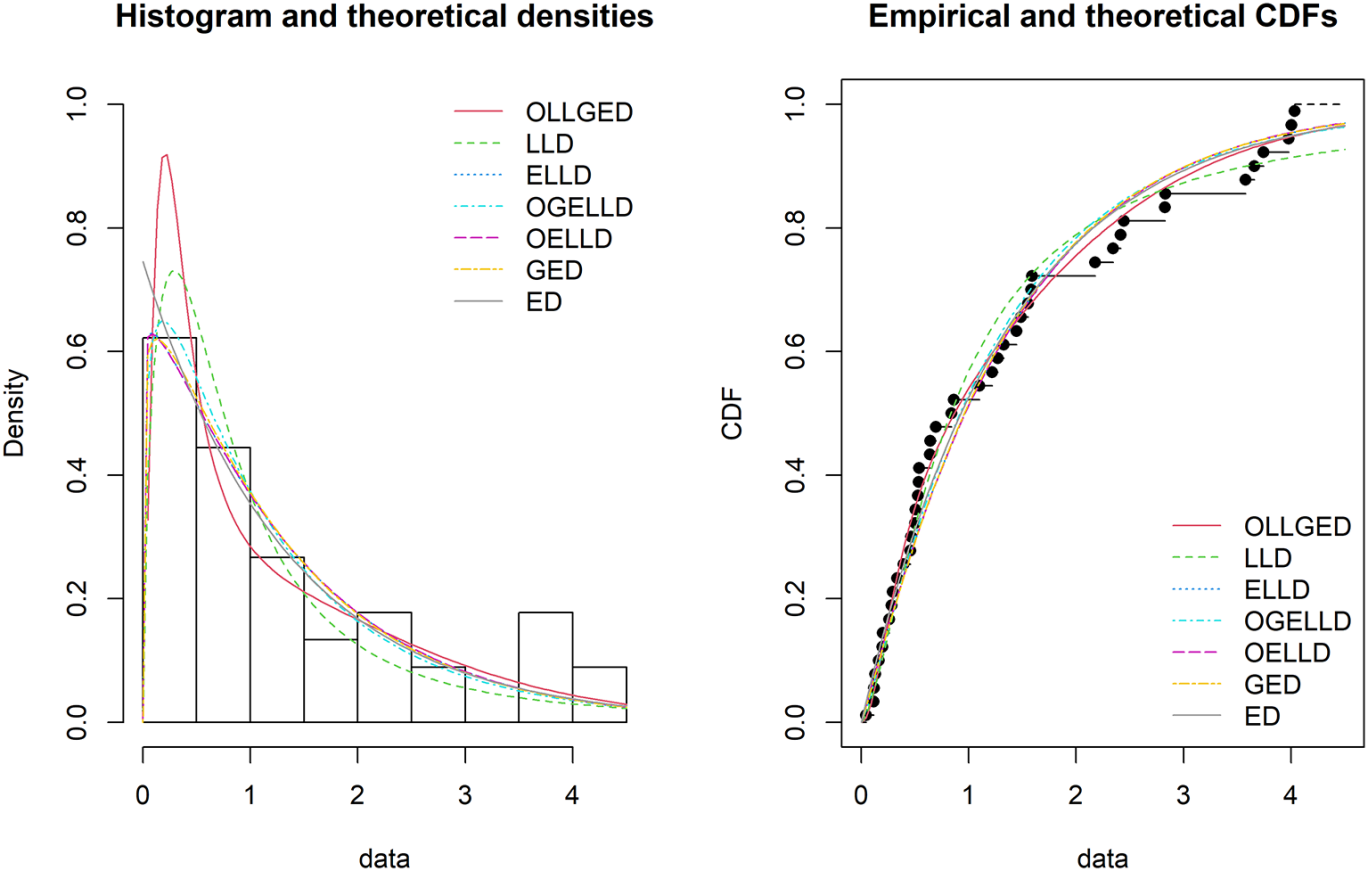
The densities fitted (left) and CDF plots (right) for various models for survival time data.

Tables 3 and 5 presented the MLEs of the model parameters respectively (of the fitted distribution) and their standard errors (SEs), KS, and *p*-value statistics for the distributions fitted OLLGED, OGELLD, OELLD, ELLD, LLD, GED, and ED models for the two data sets correspondingly. Tables 4 and 6 show the values of $-2\hat{l}$ , A ^*^ *,* W ^*^ *, BIC, and AIC* the for the two data sets separately. As shown in Tables 3- 6, the OLLGED is the best model because it has values that are the lowest for all goodness-of-fit statistics among all distributions fitted. The histogram of the first data set, fitted PDFs of the best seven fitted OLLGED, OGELLD, OELLD, ELLD, LLD, GED, and ED and their CDF plots are demonstrated in Figure 7. The histogram of the first data set fitted PDFs of the best seven fitted OLLGED, OGELLD, OELLD, ELLD, LLD, GED, and ED, and their CDF plots are displayed in Figure 8.

**Table 3.** Goodness-of-fit statistics for the waiting times' data.

Model	$-2\hat{l}$	AIC	BIC	W ^*^	A ^*^
**OLLGED(γ,α,λ)**	**579.4480**	**585.4480**	**591.9247**	**0.0982**	**0.5837**
OGELLD(α,λ,γ,σ)	587.9068	595.9068	604.5423	0.1198	0.8623
OELLD(α,λ,σ)	593.8000	598.8003	606.2791	0.1268	0.9163
ELLD(α,λ,σ)	598.5400	604.5390	611.0160	0.5723	2.9311
LLD(α,λ)	593.1488	597.1500	601.4700	0.1192	0.8909
GED(α,λ)	591.3320	595.3321	599.6498	0.1425	0.9606
ED(λ)	599.6254	601.6254	603.7843	0.1900	1.5899

**Table 4.** The estimates (SEs’), their *p*-value, and KS for waiting time data.

Model	Estimates (SEs)				KS	*p*-value
**OLLGED(γ,α,λ)**	**0.2159 (0.0225)**	**41.5710 (1.1481)**	**0.1657 (0.0134)**		**0.0967**	**0.5867**
OGELLD(α,λ,γ,σ)	32.7316 (89.1696)	0.3510 (0.2381)	0.6306 (1.6332)	2.1699 (15.6115)	0.0954	0.5604
OELLD(α,λ,σ)	1.2742 (0.1203)	5.4925 (42.2572)	11.3491 (68.5411)		0.1112	0.4072
ELLD(α,λ,σ)	0.0311 (0.0214)	24.2629 (16.0204)	7.5672 (0.3724)		0.1883	0.214
LLD(α,λ)	28.3417 (3.2940)	1.9650 (0.1985)			0.0999	0.5449
GED(α,λ)	1.7309 (0.3195)	0.0349 (0.0051)			0.1225	0.2917
ED(λ)	0.2510 (0.0310)				0.1664	0.0579

**Table 5.** Goodness-of-fit statistics for the survival time data.

Model	$-2\hat{l}$	AIC	BIC	W ^*^	A ^*^
**OLLGED(γ,α,λ)**	**112.1880**	**118.1887**	**123.6081**	**0.0289**	**0.2410**
OGELLD(α,λ,γ,σ)	116.0872	124.0872	131.3139	0.0719	0.4824
OELLD(α,λ,σ)	116.2474	122.2474	127.6674	0.0814	0.5363
ELLD(α,λ,σ)	118.2510	122.2511	127.6710	0.0812	0.5429
LLD(α,λ)	120.3712	124.3713	127.9846	0.0692	0.5028
GED(α,λ)	116.1897	120.1897	123.803	0.0827	0.5397
ED(λ)	116.4372	118.4372	124.2439	0.0589	0.4454

**Table 6.** The estimates (their SEs in parentheses), KS, and its *p*-value for survival time data.

Model	Estimates (SEs)				KS	*p*-value
**OLLGED(γ,α,λ)**	**0.3193 (0.1349)**	**7.2769 (5.3527)**	**2.7667 (1.1212)**		**0.0594**	**0.9946**
OGELLD(α,λ,γ,σ)	1.6103 (1.9739)	0.8079 (0.5274)	0.2539 (2.2143)	4.7280 (51.0672)	0.1004	0.7169
OELLD(α,λ,σ)	1.0531 (1.1238)	2.9579 (142.6629)	0.4892 (22.4021)		0.1094	0.6153
ELLD(α,λ,σ)	1261.1663 (2975.6993)	1.0505 (0.1224)	1224.1899 (2656.7019)		0.1090	0.6196
LLD(α,λ)	1.5075 (0.1832)	0.8332 (0.1460)			0.0849	0.8745
GED(α,λ)	1.1049 (1.2196)	1.7943 (1.1511)			0.1099	0.6093
ED( *λ*)	0.7455 (0.4111)				0.0908	0.8192

## 7. Discussions and conclusion

This article extends a new odd log-logistic generalized exponential distribution with three parameters to study the nature of the distribution in terms of kurtosis and skewness. The special models of the odd log-logistic generalized exponential family namely generalized exponential distribution, log-logistic distribution, and exponential distribution are presented. The common mathematical properties are obtained for the OLLGED. The parameters estimation is considered by the maximum-likelihood approach and simulation results are acquired to confirm the performance of these estimators. The application and flexibility of the OLLGED are ensured through empirical observation using two sets of lifetime data, establishing that the proposed OLLGED can provide a better fit in comparison to existing rival models, such as odd generalized log-logistic, type-II generalized log-logistic, exponential distributions, odd exponential log-logistic, generalized exponential, and log-logistic. The bias and mean square error of the parameters decrease as the sample size increases. The limitation of the proposed model is for very small values the bias and MSE are not stable. This model may not suitable for small samples and high peaked data.

### Ethical considerations

This study was based on published data, so ethical approval was not required for published data.

### Patient consent

Patient consent was not applicable as the study was based on published data.

## Consent for publication

All authors agreed to publish this paper.

## Data Availability

The first data set related to waiting times (in seconds) between 65 successive eruptions of water through a hole in the cliff at the coastal town of Kiama obtained from http://www.statsci.org/data/oz/kiama.html. Used by Silva, R., Gomes-Silva, F., Ramos, M., Cordeiro, G., Marinho, P., & Andrade, T. A. N. D. (2019). The Exponentiated Kumaraswamy-G Class: General Properties and Application. *Revista Colombiana de Estadística, 42*, 1-33. The second data set is drawn from Alizadeh, M., Tahir, M. H., Cordeiro, G. M., Mansoor, M., Zubair, M., & Hamedani, G. G. (2015). The Kumaraswamy Marshal-Olkin family of distributions. *Journal of the Egyptian Mathematical Society, 23*(3), 546-557. doi: https://doi.org/10.1016/j.joems.2014.12.002 Also, used by Bekker, A., Roux, J. J. J., & Mosteit, P. J. (2000). A generalization of the compound rayleigh distribution: using a bayesian method on cancer survival times. *Communications in Statistics - Theory and Methods, 29*(7), 1419-1433. doi: https://doi.org/10.1080/03610920008832554
