# Supplementary material for: The odd log-logistic generalized exponential distribution: Application on survival times of chemotherapy patients data
Source: F1000Res. 2023 Nov 21;11:1444. Originally published 2022 Dec 6. [Version 3] doi: 10.12688/f1000research.127363.3 (PMC11143405; doi:10.12688/f1000research.127363.3)
Supplement: Peer review history

# Reviewer report: Reviewer response for version 3

**DOI:** 10.5256/f1000research.158863.r224409

**Published:** 2024-05-31

**Authors:** Sadaf Khan

**Affiliations:** 1 The islamia university bahawalpur, Bahawalpur, Pakistan

Accept

Is the work clearly and accurately presented and does it cite the current literature?

Yes

If applicable, is the statistical analysis and its interpretation appropriate?

Yes

Are all the source data underlying the results available to ensure full reproducibility?

Yes

Is the study design appropriate and is the work technically sound?

Partly

Are the conclusions drawn adequately supported by the results?

Partly

Are sufficient details of methods and analysis provided to allow replication by others?

Partly

Reviewer Expertise:

Distribution theory, applied statistics, mathematical modelling.

I confirm that I have read this submission and believe that I have an appropriate level of expertise to confirm that it is of an acceptable scientific standard.

# Reviewer report: Reviewer response for version 2

**DOI:** 10.5256/f1000research.153883.r195521

**Published:** 2023-11-10

**Authors:** Sadaf Khan

**Affiliations:** 1 The islamia university bahawalpur, Bahawalpur, Pakistan

I did not find left skewness in Figure 1 of the proposed model.

 The remarks 4,5,8,9 of review round1 have not been addressed in a satisfactory manner.

Is the work clearly and accurately presented and does it cite the current literature?

Yes

If applicable, is the statistical analysis and its interpretation appropriate?

Yes

Are all the source data underlying the results available to ensure full reproducibility?

Yes

Is the study design appropriate and is the work technically sound?

Partly

Are the conclusions drawn adequately supported by the results?

Partly

Are sufficient details of methods and analysis provided to allow replication by others?

Partly

Reviewer Expertise:

Distribution theory, applied statistics, mathematical modelling.

I confirm that I have read this submission and believe that I have an appropriate level of expertise to confirm that it is of an acceptable scientific standard, however I have significant reservations, as outlined above.

# Response

**Published:** 2023-11-11

**Authors:** Srinivasa Rao Gadde

**Affiliations:** The University of Dodoma, Tanzania

Dear Editor-in-Chief and Reviewer:

 Thank you very much for giving us the opportunity to revise our manuscript. We also appreciate the valuable input of anonymous reviewers. We are so happy that all the reviewers have given positive feedback and encouraged the publication of our paper.

 The suggestions made by all reviewers are incorporated, and changes are highlighted in the revised manuscript with RED color. Below are the responses regarding the comments made by the respected reviewer. We hope the revised manuscript will justify the higher standards of the journal.

**Comments and Responses**

**Comment: **I did not find left skewness in Figure 1 of the proposed model.

**Response: **Thank you for pointing out the mistake; we have deleted the word left skewed in the revised manuscript. We have tried many combinations of parameters, but unfortunately, the shapes are appearing in this manner. 

**Comment: **The remarks 4,5,8,9 of review round 1 have not been addressed in a satisfactory manner.

**Response: **Respected sir, we are extremely sorry for the confusion and for not addressing those comments well.

**Response to comment 4:** The nature of distributions changes with the change in shape parameters.  This was explained after drawing Figure 1, and now we have provided some more explanation on the nature of the distribution.

**Response to comment 5: We have provided some disadvantages of our model.**

**Response to comment 8: **Figures 7 and 8 are reconstructed by adding PP plots. In the literature review, numerous authors have displayed the figures (Figures 7 and 8) for comparing the various models. Due to this reason, we have displayed only pdf and CDF plots. As for your suggestion, we have added one more figure as a PP-plot. When we are fitting a single model for data, more researchers display it in pdf, cdf, pp, and qq plots. I hope the figures are now up to your expectations. Please refer to the following references: We have provided high-resolution figures.

 Ishaq AI, Suleiman AA, Daud H, Singh NSS, Othman M, Sokkalingam R, Wiratchotisatian P,

 Usman AG and Abba SI (2023)  Log-Kumaraswamy distribution: its features and applications. *Front. Appl. Math. Stat. *9:1258961. doi: 10.3389/fams.2023.1258961

 Shah, S., Hazarika , P. J., Chakraborty , S., & Alizadeh, M. (2022). The Balakrishnan-Alpha-Beta-Skew-Laplace Distribution: Properties and Applications. *Statistics, Optimization & Information Computing*, *11*(3), 755-772. https://doi.org/10.19139/soic-2310-5070-1247

 Akarawak, E. E. E., Adeyeye, S. J., Khaleel, M. A., Adedotun, A. F., Ogunsanya, A. S., & Amalare, A. A. (2023). The inverted Gompertz-Fréchet distribution with applications. *Scientific African, 21*, e01769. doi: https://doi.org/10.1016/j.sciaf.2023.e01769

 Shah, S., Hazarika, P. J., Chakraborty, S., & Ali, M. M. (2021). The Balakrishnan-Alpha-Beta-Skew-Normal Distribution: Properties and Applications. *Pakistan Journal of Statistics and Operation Research*, *17*(2), 367-380. https://doi.org/10.18187/pjsor.v17i2.3731

**Response to comment 9: Thank you for your suggestion; we have added some more explanation.**

# Reviewer report: Reviewer response for version 2

**DOI:** 10.5256/f1000research.153883.r195520

**Published:** 2023-08-17

**Authors:** Boikanyo Makubate

**Affiliations:** 1 Botswana International University of Science and Technology, Palapye, Central District, Botswana

The manuscript has improved. The manuscript is scientifically valid in its current form. I recommend the manuscript for indexing.

Is the work clearly and accurately presented and does it cite the current literature?

No

If applicable, is the statistical analysis and its interpretation appropriate?

No

Are all the source data underlying the results available to ensure full reproducibility?

No

Is the study design appropriate and is the work technically sound?

No

Are the conclusions drawn adequately supported by the results?

No

Are sufficient details of methods and analysis provided to allow replication by others?

No

Reviewer Expertise:

Mathematical Statistics, Distributional theory, Applied Statistics, Medical statistics

I confirm that I have read this submission and believe that I have an appropriate level of expertise to confirm that it is of an acceptable scientific standard.

# Reviewer report: Reviewer response for version 1

**DOI:** 10.5256/f1000research.139863.r171415

**Published:** 2023-07-06

**Authors:** Boikanyo Makubate

**Affiliations:** 1 Botswana International University of Science and Technology, Palapye, Central District, Botswana

The comments on the above-mentioned manuscript are as follows:

1. Although some of the paper results seem correct, I have some doubts about whether there is enough innovation for a new publication, especially as many other related papers have been published on this topic. Could you justify it more convincingly?
2. In addition the previous item, I would like to have read more tenable arguments about the need for a new distribution. Otherwise, you are left with just an analytical exercise of a new distribution that may never be used in practice. Would you be able to revise the text of the article in order to better justify it and thus make it more interesting?
3. Section 3.1 and hence 3.2 are incorrect.
4. I expected to see more discussion in the simulation study, real data analysis and conclusion.
5. I could not find detailed information about the software used for obtaining the results of simulation study and real data analysis.  
6. Lastly, I also suggest reviewing the use of English carefully, and considerable rewording and pruning to make the paper more concise and precise.
7. Fig 1, the pdf plots, the researcher should include left skewed and symmetric shapes as they are explained in page 4.
8. Skewness plot on fig 5 is not well explained in relation to the pdf plots, especially the area of the graph in which we can observe symmetric, right or left skewness.
9. The kurtosis on Fig 6, is not interpreted especially in relation to leptokurtic, mesokurtic and platykurtic, and which area of the graph we can observe these.
10. On section 4, the researcher mentioned that they used the MLE parameter estimation technique as it is commonly used in the literature, the researcher should justify the method in relation to its efficiency, consistency, asymptotically normal and invariant property under transformation.
11. On section 6, the OLLGED has 3 parameters, LLD and GED have 2 parameters and the ED has a single parameter, therefore their comparison is nor fair based on the number of parameters. Suggested is to include at least 3 more 3 parameter comparative models. Interpretation of Fig 7 and 8 have to be incorporated
12. Finally,  I do not recommend this article for indexing. It is poorly written and contains a lot of incorrect results.

Is the work clearly and accurately presented and does it cite the current literature?

No

If applicable, is the statistical analysis and its interpretation appropriate?

No

Are all the source data underlying the results available to ensure full reproducibility?

No

Is the study design appropriate and is the work technically sound?

No

Are the conclusions drawn adequately supported by the results?

No

Are sufficient details of methods and analysis provided to allow replication by others?

No

Reviewer Expertise:

Mathematical Statistics, Distributional theory, Applied Statistics, Medical statistics

I confirm that I have read this submission and believe that I have an appropriate level of expertise to state that I do not consider it to be of an acceptable scientific standard, for reasons outlined above.

# Response

**Published:** 2023-07-29

**Authors:** Srinivasa Rao Gadde

**Affiliations:** The University of Dodoma, Tanzania

1. Although some of the paper results seem correct, I have some doubts about whether there is enough innovation for a new publication, especially as many other related papers have been published on this topic. Could you justify it more convincingly?

 2. In addition, the previous item, I would like to have read more tenable arguments about the need for a new distribution. Otherwise, you are left with just an analytical exercise of a new distribution that may never be used in practice. Would you be able to revise the text of the article in order to better justify it and thus make it more interesting?

**Response:** The motive for extending distributions for modelling lifetime data is the capacity to simulate both monotonically and non-monotonically growing, decreasing, and constant failure rates, or more critically with bathtub-shaped failure rates, even if the baseline failure rate is monotonic. The fundamental justifications for implementing a new distribution model in practice are as follows: to create tail weight distributions for modelling various real data sets, to generate distributions with negative, positive, and symmetric skewness, to define special models with all varieties of hazard rate functions, to make the kurtosis more flexible than the baseline distribution, and to consistently produce better fits than other generated distributions with the same underlying model.

 3. Section 3.1 and hence 3.2 are incorrect

**Response: **Some typographical errors were corrected

 4. I could not find detailed information about the software used for obtaining the results of simulation study and real data analysis

**Response: **Explanation on the software used for various analytical activities in this paper is given in last part of section 4

 5. I expected to see more discussion in the simulation study, real data analysis and conclusion

**Response: **More discussion on simulation is given in section 5 on the simulation and explanation of the so obtained results. Discussion on the real data used for the application is made in section 7.

 6.  On section 6, the OLLGED has 3 parameters, LLD and GED have 2 parameters and the ED has a single parameter, therefore their comparison is nor fair based on the number of parameters. Suggested is to include at least 3 more 3 parameter comparative models. Interpretation of Fig 7 and 8 have to be incorporated

**Response: **The suggested was compared with the commonly used distribution regardless of the number of parameters for there were distributions with four parameters see Table 3 proposed distribution proved to be more powerful over them, those with fewer distributions were used too to show that the proposed distribution would stand them too. 

 7.  Lastly, I also suggest reviewing the use of English carefully, and considerable rewording and pruning to make the paper more concise and precise

**Response: **There manuscript was taken to the editor to structure it well the grammar

 8.  Fig 1, the pdf plots, the researcher should include left skewed and symmetric shapes as they are explained on page 4.

**Response: **The fourth part of Figure 1 shows the left-skewed and symmetric shapes.

 9. The kurtosis on Fig 6, is not interpreted especially in relation to leptokurtic, mesokurtic and platykurtic, and which area of the graph we can observe these.

**Response: **When we fix the parameter *λ* , the skewness and kurtosis of OLLGED increases as *α*  and *γ*  increases. More specifically when parametric values are increased the skewness becomes negative and kurtosis becomes mesokurtic.  

 10.  On section 4, the researcher mentioned that they used the MLE parameter estimation technique as it is commonly used in the literature, the researcher should justify the method in relation to its efficiency, consistency, asymptotically normal and invariant property under transformation.

**Response: **The justification is given for using MLE as suggested

 11. On section 6, the OLLGED has 3 parameters, LLD and GED have 2 parameters and the ED has a single parameter, therefore their comparison is nor fair based on the number of parameters. Suggested is to include at least 3 more 3-parameter comparative models. Interpretation of Fig 7 and 8 have to be incorporated

**Response: **The suggested was compared with the commonly used distribution regardless of the number of parameters for there were distributions with four parameters see table 3 proposed distribution proved to be more powerful over them, those with fewer distributions were used too to show that the proposed distribution would stand them too.

# Reviewer report: Reviewer response for version 1

**DOI:** 10.5256/f1000research.139863.r181917

**Published:** 2023-07-06

**Authors:** Sadaf Khan

**Affiliations:** 1 The islamia university bahawalpur, Bahawalpur, Pakistan

In this article, the odd log-logistic generalized exponential distribution (OLLGED) is proposed using Odd Log Logistic-G family, originally proposed by Gleaton and Lynch (2006). Various statistical properties including generating functions, moments, quantile and order statistics are dervied mathematically. The estimation of the model parameter is achieved by the maximum likelihood method and related inferences have been drawn. The convergence of the parameters estimates has been verified by Monte-Carlo simulation methods. The model is compared with 6 well established distributions by applying it on two real data sets.​​​​

1. Figure 1: bottom left and right are same.
2. Motivations are less convincing. There is no novelty in the proposed work. The authors need to justify why they chose generalize exponential distribution as baseline to OLL-G proposed by Gleaton and Lynch (2006) ^1^.
3. How do the authors justify the proposed distribution utility and scope in various scientific fields?
4. How come the authors justify the shape parameters’ physical interpretation?
5. What are some possible disadvantages of the model given in (5) and (6)?
6. In section 7, I would also recommend that the author comment more on the limitation of this distribution. How the distribution change when the size is large or too small, for example. Is it a critical point where the distribution does not apply anymore?
7. In section 7, the authors did not show how to interpret model parameters and their practical meanings in real data. How does OLLGED perform in comparison to OLL-Gamma, OLL-Weibull or OLL-LogNormal distribution?
8. Figure 7 and Figure 8 are difficult to read. Also add other graphical measures such as PP-Plots, QQ-Plots, estimated hazard rate etc.
9. Comment on the behavior of the datasets being employed.
10. What are future directives one can extract from the proposed model?

Is the work clearly and accurately presented and does it cite the current literature?

Yes

If applicable, is the statistical analysis and its interpretation appropriate?

Yes

Are all the source data underlying the results available to ensure full reproducibility?

Yes

Is the study design appropriate and is the work technically sound?

Partly

Are the conclusions drawn adequately supported by the results?

Partly

Are sufficient details of methods and analysis provided to allow replication by others?

Partly

Reviewer Expertise:

Distribution theory, applied statistics, mathematical modelling.

I confirm that I have read this submission and believe that I have an appropriate level of expertise to confirm that it is of an acceptable scientific standard, however I have significant reservations, as outlined above.

## References

[1] : Properties of generalized log-logistic families of lifetime distributions. *J Probab Statist Sci.* .2006;4(1) :51-64

# Response

**Published:** 2023-07-29

**Authors:** Srinivasa Rao Gadde

**Affiliations:** The University of Dodoma, Tanzania

**Reviewer Report 1**

 1 Figure 1: bottom left and right are same

**Response**: The two figures are not the same see the shape parameter sigma which made the change in the figures

 2. Motivations are less convincing. There is no novelty in the proposed work. The authors need to justify why they chose generalize exponential distribution as baseline to OLL-G proposed by Gleaton and Lynch (2006)1.

**Response:** More justification for the selection of GED as baseline over others distributions in existence including OLL-G as proposed by Gleaton and Lynch (2006)

 3 How do the authors justify the proposed distribution utility and scope in various scientific fields?

**Response:** A number (6) of justification for the proposed distribution are given

 4. How come the authors justify the shape parameters’ physical interpretation?

**Response**: We have explained the various physical appearances of our model in Figure 1

 5. What are some possible disadvantages of the model given in (5) and (6)?

**Response:** They are very flexible and change into other distributions as the parameters change

 6. In section 7, I would also recommend that the author comment more on the limitation of this distribution. How the distribution change when the size is large or too small, for example? Is it a critical point where the distribution does not apply anymore?

**Response:** The bias and mean square error of the parameters decrease as the sample size increases. The limitation of the proposed model is for very small values the bias and MSE are not stable. This model may not suitable for small samples and high peaked data.

 7. In section 7, the authors did not show how to interpret model parameters and their practical meanings in real data. How does OLLGED perform in comparison to OLL-Gamma, OLL-Weibull or OLL-Log Normal distribution?

**Response:** The part was added to give more practical meaning and comparison of suggested OLLGED to other existing distributions.

 8. Figure 7 and Figure 8 are difficult to read. Also add other graphical measures such as PP-Plots, QQ-Plots, estimated hazard rate etc.

**Response:** Now figures can be ready and pp and qq are displayed in Figure 9 and Figure 10

 9.  Comment on the behavior of the datasets being employed.

**Response:** Table 3 and Table 6 have been added to describe data sets

 10. What are future directives one can extract from the proposed model?

**Response:** The proposed OLLGED is among the best in dealing with heavy-tailed data sets and right-skewed data
